# *Cryptococcus* displays spore-specific uptake by alveolar epithelial cells

**DOI:** 10.1128/mbio.01831-25

**Published:** 2025-10-22

**Authors:** Sébastien C. Ortiz, Rachael Fortune-Grant, Andrew J. Thom, Joshua Davies, Robin C. May, Rebecca A. Drummond, Margherita Bertuzzi

**Affiliations:** 1 Manchester Fungal Infection Group, Faculty of Biology, Medicine and Health, The University of Manchester, Core Technology Facility 12203, Manchester, United Kingdom; 2 Institute of Microbiology & Infection and School of Biosciences, University of Birmingham 85442, Birmingham, United Kingdom; 3 Institute of Immunology & Immunotherapy, Institute of Microbiology & Infection, University of Birmingham 152871, Birmingham, United Kingdom; The University of Georgia, Athens, Georgia, USA

**Keywords:** *Cryptococcus neoformans*, *C. deneoformans*, spores, airway epithelial cells, phagocytosis, fungal pathogenesis, latency, dissemination

## Abstract

Human fungal pathogens, including *Cryptococcus neoformans,* cause 1.5 million annual deaths. *Cryptococcus* causes disease when it disseminates out of the lung and into the brain, which can occur years after initial exposure (latency) via mechanisms that remain unknown. Spores of *Cryptococcus* display distinct surface epitopes, host interactions, and disease kinetics to the vegetatively growing yeast morphotype, yet they remain understudied, likely contributing to our lack of understanding of pathogenesis. One of the first barriers spores encounter is non-professional phagocytic airway epithelial cells (AECs). Here, we demonstrate that *Cryptococcus* spores are preferentially internalized by AECs both *in vitro* and *in vivo*. Once inside, spores can germinate, subsequently replicate, persist, and/or escape. This ability to enter AECs correlates with a preferential ability of spores to cross AEC barriers. Together, our work indicates that AECs internalize *Cryptococcus* spores and may serve as a previously ignored intracellular host niche, providing alternative hypotheses for both *Cryptococcus* dissemination and latency.

**IMPORTANCE:**

Fungal spores are a dormant, stress-resistant, and relatively understudied cell type and are presumed infectious cell types in cryptococcal disease. *Cryptococcus* spores have been shown to display distinct disease kinetics to the vegetative yeast morphotype and are significantly better at disseminating out of the host lung. While the molecular mechanisms by which spores disseminate out of the lung have yet to be identified, their preferential ability to get inside host cells likely enables their dissemination. Here, we show that spores, unlike yeast, readily get taken up by non-professional phagocytic cells, airway epithelial cells, both *in vitro* and *in vivo,* and once inside can germinate, replicate, escape, and/or persist. These results provide a previously unexplored host cell type that *Cryptococcus* can inhabit, likely affecting disease kinetics and could be a critical interaction in understanding both extrapulmonary dissemination and latency.

## INTRODUCTION

Invasive fungal diseases cause over 1.5 million deaths annually, with mortality rates often over 50% due to lack of diagnostics and antifungal treatments (1, 2). By understanding how fungi establish infections, persist in hosts, and disseminate, we can develop much-needed diagnostics, prevention, and antifungal therapeutics to combat invasive fungal disease. *Cryptococcus neoformans,* an inhaled facultative intracellular pathogen, is at the top of the WHO’s priority list of human fungal pathogens, largely due to the high mortality associated with cryptococcosis, resulting in an estimated 181,000 annual deaths (3, 4). Key aspects of *Cryptococcus* disease progression are still not understood. Studies have focused on understanding the virulence of this pathogen and its ability to colonize the brain (the primary site of disease), but little is known about how *Cryptococcus* disseminates out of the lung (the initial site of infection). Additionally, via unknown mechanisms, *Cryptococcus* undergoes latency and causes disease in hosts years or decades after initial infection (5, 6). Accordingly, after treatment for cryptococcosis, patients living with HIV/AIDS undergo a minimum of a year of secondary prophylaxis to prevent resurgence (7). The mechanisms of *Cryptococcus* dissemination, latency, and persistence remain unknown.

One of the potential reasons that the pathogenesis of *Cryptococcus* remains poorly understood is that the morphotype used for the vast majority of studies is the yeast form; however, yeasts are too large to reach the lower airway and thus unlikely to initiate infection under normal circumstances. *Cryptococcus* produces sexual spores, a dormant and stress-resistant morphotype small enough to reach lower airways; consequently, spores are the presumed infectious morphotype in *Cryptococcus* disease (8, 9). Alas, difficulties associated with producing and working with *Cryptococcus* spores have resulted in a problematic knowledge gap regarding spore-mediated infections, despite spores having distinct surface properties, interacting with host immune cells differently than yeast, and, critically, disseminating out of the lung when yeast cannot (9–13). The mechanisms of spore-specific dissemination remain unknown, emphasizing a need for the investigation of spore pathogenesis (13).

One of the first barriers that inhaled pathogens encounter is non-professional phagocytic airway epithelial cells (AECs). Alveolar AECs make up 24% of the lung parenchyma, likely have extensive and prolonged contact with inhaled fungal spores, and are key for the antimicrobial lung defense, which if misregulated may lead to pathogen persistence (14–17). Studies looking at the interactions of *Cryptococcus* with AECs show that yeast are not readily internalized (18–21). Given the unique characteristics of spores, we hypothesized that spores interact differently with AECs compared to yeast and found that *Cryptococcus* spores are internalized by AECs both *in vitro* and *in vivo*. Once inside, spores can germinate, replicate, persist, and even escape. Furthermore, spores show preferential crossing of epithelial barriers, potentially implicating spore-AEC interactions in dissemination out of the lung. Our results identify AECs as a novel host cell type that *Cryptococcus* can occupy and exploit in a spore-specific manner. AECs also provide an intracellular environment that may facilitate latency/persistence of spores, a fundamentally dormant morphotype. These results provide viable alternative hypotheses to *Cryptococcus* dissemination, latency, and persistence and emphasize the importance of evaluating different morphotypes in pathogenesis to open the door to novel opportunities for much-needed diagnostic and therapeutic intervention.

## RESULTS

### *Cryptococcus* spores, unlike yeast, are readily internalized by AECs and can germinate and replicate intracellularly

Previous studies investigating the ability of *Cryptococcus* yeast to enter AECs have shown that even at high infectious doses (multiplicity of infection [MOI] of 10), very few yeast cells are internalized (20). Internalization increases slightly for acapsular mutants, but these are avirulent and thus unlikely to be a cause of human infection (18–21). Spores are known to have distinct surface properties than yeast cells, suggesting that they may interact with AECs differently (9–11, 22).

**Fig 1:**
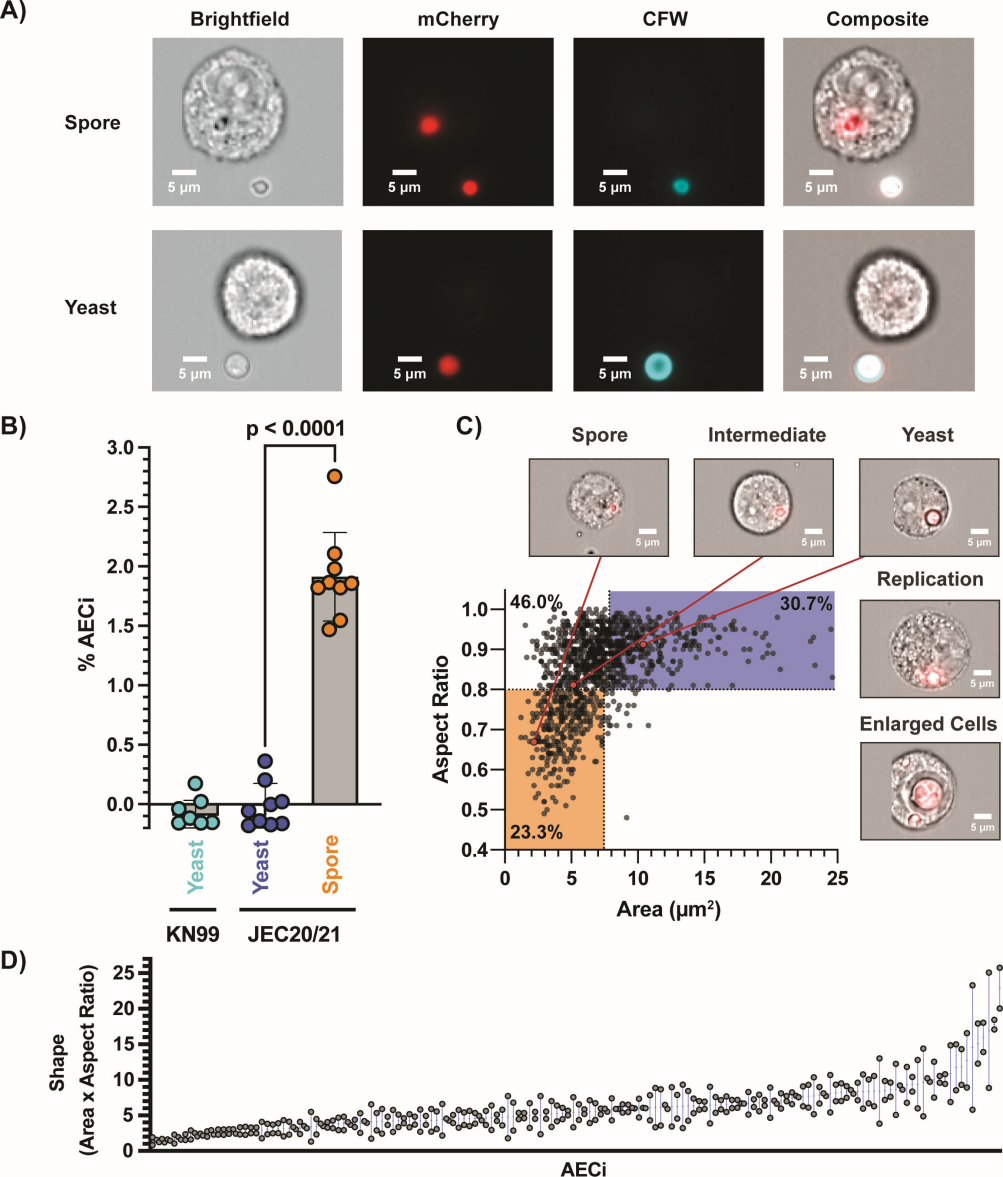
Spore-specific uptake of *Cryptococcus* by airway epithelial cells (AECs). (**A**) Representative images from IFC, 6 hpi. mCherry-tagged *Cryptococcus* used to identify AECs interacting fungal cells, and calcofluor white, which will only stain external *Cryptococcus,* was used to distinguish internalized from non-internalized *Cryptococcus*. (**B**) Percentage of AECs with internalization events (AEC_i_) quantified for yeast (KN99 and JEC20+21), and spores (JEC20×JEC21), demonstrating that spores are readily internalized while yeast are not. A value of 1.91% AEC_i_ reflects 15.4% of spore infectious dose being internalized. One-way analysis of variance, *P* < 0.0001, (**C**) IFC was performed 24 hours post-infection to evaluate intracellular germination kinetics. Morphotypes were quantified based on cell size (surface area, µm^2^) and shape (aspect ratio), with spores being small and oval and yeast being large and round. Along with heterogeneous germination kinetics, both intracellular replication and cell enlargement were observed. (**D**) Correlation of spore shape for *Cryptococcus* residing within the same AECs (stoichiometry = 2) demonstrates a link between host AEC and germination kinetics. Two-tailed Spearman correlation analysis, *r* = 0.5219.

To test this, we exposed AECs to spores and carried out differential staining followed by imaging flow cytometry (IFC) (Fig. 1) (23). A549 immortalized type II alveolar epithelial cells were exposed to either mCherry (mCh)-tagged *Cryptococcus* spores (produced from prolific sporulator strains JEC20×JEC21), yeast of the same background (JEC20+21), or yeast of the previously evaluated background (KN99α) at a low MOI (0.167). Internalization was evaluated 6 hours post-infection (hpi), and counterstaining with calcofluor white (CFW) was used to differentiate between AECs associated with *Cryptococcus* extracellularly versus AECs containing intracellular *Cryptococcus* (AEC_i_) (Fig. 1A). Consistent with previous studies, neither KN99α nor JEC20+21 yeast cells were taken up by AECs; however, JEC20×21 spores were readily internalized by AECs with 1.91% of AECs having one or more internalized spores (1.91% AEC_i_) (Fig. 1B) (20, 21). Twenty percent of occupied AECs had more than one spore per AEC with an average stoichiometry of 1.34. This rate of internalization is equivalent to ~15.4% of infectious inoculum being internalized by AECs.

To determine if the spore-specific internalization observed was due to intrinsic spore properties rather than a generic induction of host response to *Cryptococcus* spore challenge, internalization was evaluated during a 6 hour co-infection with spores and yeast cells with different fluorophores (mCh and green fluorescent protein [GFP]) (Fig. S1A). These experiments show that during spore/yeast co-infections, regardless of the fluorophore utilized, only spores can occupy AECs. Spores tested are produced from congenic strain pairs and therefore have a homogeneous genotype; however, to ensure that the observed differences in internalization were due to morphotype-specific differences in the properties of spores/yeast cells, rather than the result of a recombinant population, JEC20×21 spores were germinated into yeast (JEC20×21 yeast). Upon infection of AECs, JEC20×21 yeast mirrored the lack of internalization displayed by the JEC20+21 yeast populations (Fig. S1B). These results supported that spore-specific invasion of AECs is due to intrinsic spore properties rather than a generic induction of a host response or the recombinant nature of spores. The spore-specific ability to occupy AECs was further validated in primary AECs (Fig. S1C) and with serotype A KN99“a”xα spores (Fig. S1D), confirming morphotype-specific invasion of AECs by spores but not the yeast counterpart.

In order to cause disease, spores need to germinate into vegetatively growing yeast. *Cryptococcus* spores readily germinate in the lung environment, with cryptococci recovered from the bronchial alveolar lavage of spore-infected mice 18 hours after infection having fully transitioned to the yeast form (13). In the absence of AECs, spore populations were found to germinate fully by 24 hours in tissue culture conditions (37°C + 5% CO_2_) with a previously established bimodal phenotype (Fig. S2) (24). To determine if spores germinate within AECs, differential staining and IFC was performed on cells after 24 hours of infection with either spores or yeast (MOI 1), and intracellular germination was evaluated by quantifying the size (surface area) and circularity (aspect ratio) of internalized *Cryptococcus* using previously established metrics (25). Even with this higher MOI, spores were readily internalized by AECs (12.6% AEC_i_) as opposed to yeast (0% AEC_i_) (Fig. S3Ai). At 24 hours post-infection, intracellular *Cryptococcus* was present as different morphotypes, i.e., 30.7% yeast, 46.0% intermediates, and a remaining 23.3% spores (Fig. 1C), indicating that spores readily germinate but that germination is inhibited in the intracellular environment. This population spread demonstrates a previously characterized asynchronous phenotype, indicating that internalized spores either react non-uniformly within the intracellular environment or that AECs have a range of intracellular environments that spores must germinate in (24). These possibilities are not mutually exclusive. In addition to germination state, we observed that germinated spores can actively replicate 24 hours post-infection, as evidenced by daughter cell formation. Enlarged intracellular *Cryptococcus* yeast was also observed, consistent with the large size variation that is classically observed in lung environments (26). The intracellular stoichiometry did not dictate the level of germination given that comparable germination states were observed across AEC_i_ having internalized different numbers of spores (Fig. S4A). On the other hand, when multiple spores were internalized by the same AEC_i_, intracellular spores displayed similar germination kinetics with similar shapes observed 24 hours post-infection (Fig. 1D: Stoichiometry = 2, Fig. S4B: stoichiometry ≥ 3), suggesting that germination is linked to each internalization event. Together, these results show that spores can get inside AECs and, once inside, can germinate heterogeneously and subsequently replicate. This implicates AECs as a novel spore-specific intracellular niche that *Cryptococcus* can occupy and proliferate within.

### Once germinated, *Cryptococcus* can undergo rapid replication, non-lytic escape, or persist inside AECs

**Fig 2:**
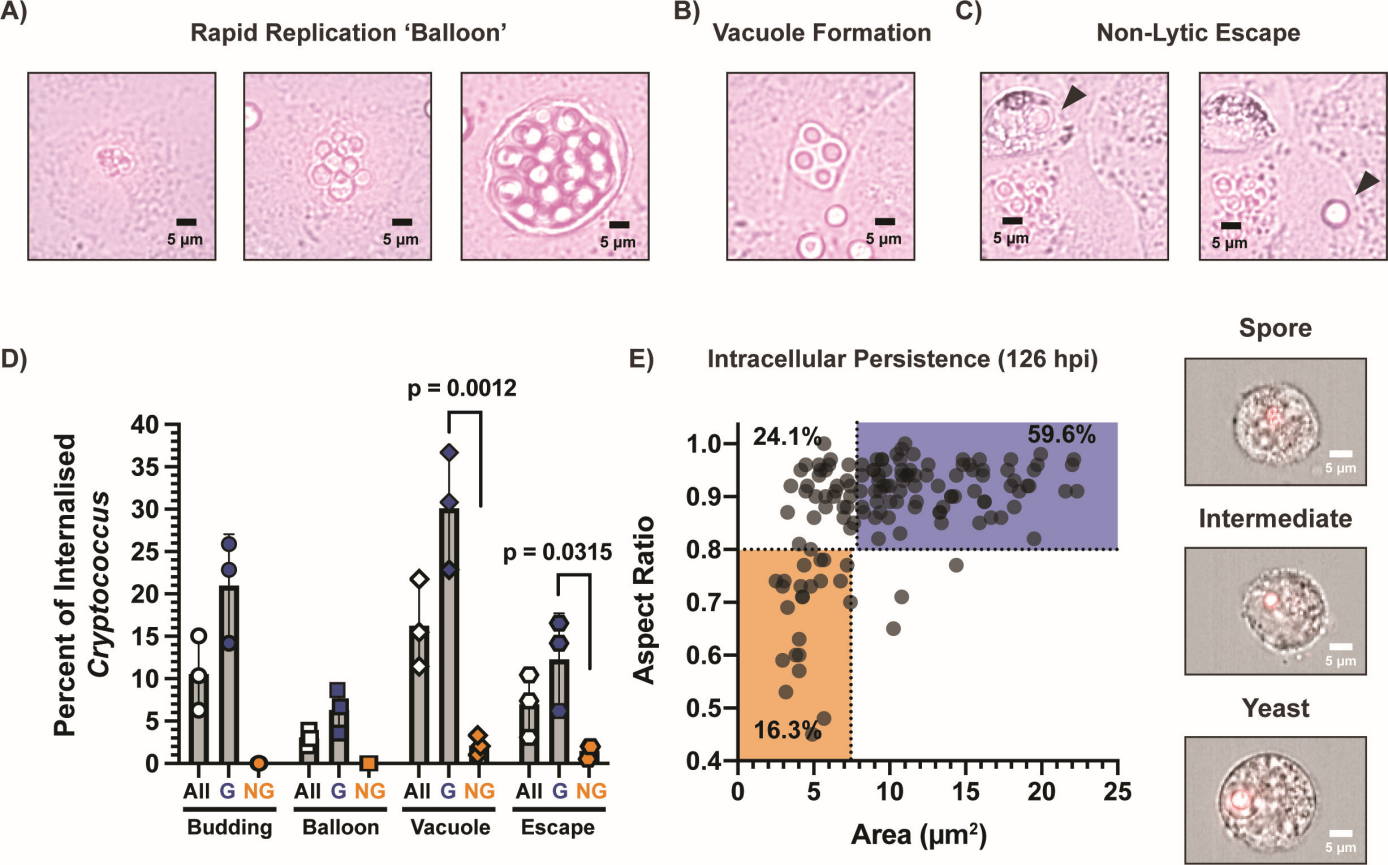
Internalized *Cryptococcus* spores (JEC20×JEC21) display a variety of behaviors. Representative images of behaviors observed using time-course microscopy of (**A**) rapid replication: internalized clusters of spores (6 hpi), efficiently germinating (16 hpi), and proceeding to rapidly replicate, resulting in an AEC full of *Cryptococcus* yeast (24 hpi). (**B**) Vacuole formation (25 hpi). (**C**) Non-lytic escape: internalized spore having germinated (24 hpi) undergoes non-lytic escape (25 hpi). (**D**) Quantification of intracellular behaviors: population percentages for all, germinated (G), or non-germinated (NG) *Cryptococcus* showing the behaviors described: budding, rapid replication, vacuole formation, and non-lytic escape. Statistics from one-way analysis of variance. (**E**) Intracellular persistence: IFC identified intracellular *Cryptococcus* in spore-infected cells 126 hpi, displaying high levels of germination and the presence of ungerminated spores and intermediates.

To further examine the behavior of internalized and germinated spores, fluorescence microscopy was used to monitor intracellular populations hourly from 6 to 26 hours post-infection. CFW counterstaining was used at 6 hours post-infection to demark internalization events. We identified three behaviors that have previously been identified for intracellular yeast in macrophages, which are rapid replication (Fig. 2A), large vacuole formation (Fig. 2B), and non-lytic escape—also termed vomocytosis (Fig. 2C) (27–29). Between 240 and 358 internalized *Cryptococcus* events per biological replicate were evaluated for the occurrence of budding, rapid replication balloon formation, vacuole formation, and non-lytic escape over 26 hours (Fig. 2D). Intracellular budding occurred in 10.6% of internalization events (21.0% of germinated spores), while rapid replication balloons occurred in 3.0% of events (6.3% of germinated spores). Vacuole formation occurred in 30.1% of germinated spores and only 2.0% of ungerminated spores (16.2% of all events). Finally, non-lytic escape was observed for 12.3% of germinated intracellular spores, and only 1.5% of non-germinated spores (7.0% of all events). These results demonstrate a large heterogeneity of intracellular behaviors and that *Cryptococcus* can readily escape AECs once germinated.

To investigate whether internalized *Cryptococcus* can persist within AECs for a prolonged period, A549 cells were infected with spores or yeast for 6 hours, then re-seeded to a low density to allow continued growth for an additional 5 days (120 hours). After 5 days, cells were evaluated for the presence of internal *Cryptococcus* cells. Despite the daily duplication of AECs, we were still able to detect intracellular *Cryptococcus* in spore-infected AECs after 126 hours of infection (Fig. S3Aii). The germination state of these internalized *Cryptococcus* cells was evaluated, and a full range of germination morphotypes was identified with 59.6% yeast, 24.1% intermediates, and a remaining 16.3% spores (Fig. 2E). While this population was more germinated than at 24 hours (up from 30.7% yeast), there were still spores and intermediates present after 126 hours. To determine the viability of intracellular *Cryptococcus* 126 hpi, internalization events were sorted using fluorescence-assisted cell sorting (FACS) and grown in liquid yeast-peptone-dextrose (YPD) for 1 week. Viability was evaluated for 864 internalization events across technical and biological triplicates (96/replicate), and the presence of fungal growth showed that 79.25% (standard deviation: 5.60) of AECs with internalization events had viable *Cryptococcus* capable of growth under these conditions. It is formally possible that *Cryptococcus* escapes these AECs and re-enters; however, non-lytic escape was rare for non-germinated spores (1.5%) unlike germinated spores (12.3%) (Fig. 2D), and we have shown that once germinated into yeast, *Cryptococcus* loses its ability to be internalized by AECs (Fig. S1B). Additionally, AECs do not show significant signs of apoptosis or necrosis after 6, 24, and 126 hours of infection, likely due to the low virulence of JEC20/JEC21, making lysis and re-entering much less likely than intracellular persistence (Fig. S3B) (30). Together, these results demonstrate that AECs provide an intracellular environment in which spores can germinate, replicate, escape, and even persist.

### *Cryptococcus* spores preferentially cross AEC barriers and are internalized *in vivo*

**Fig 3:**
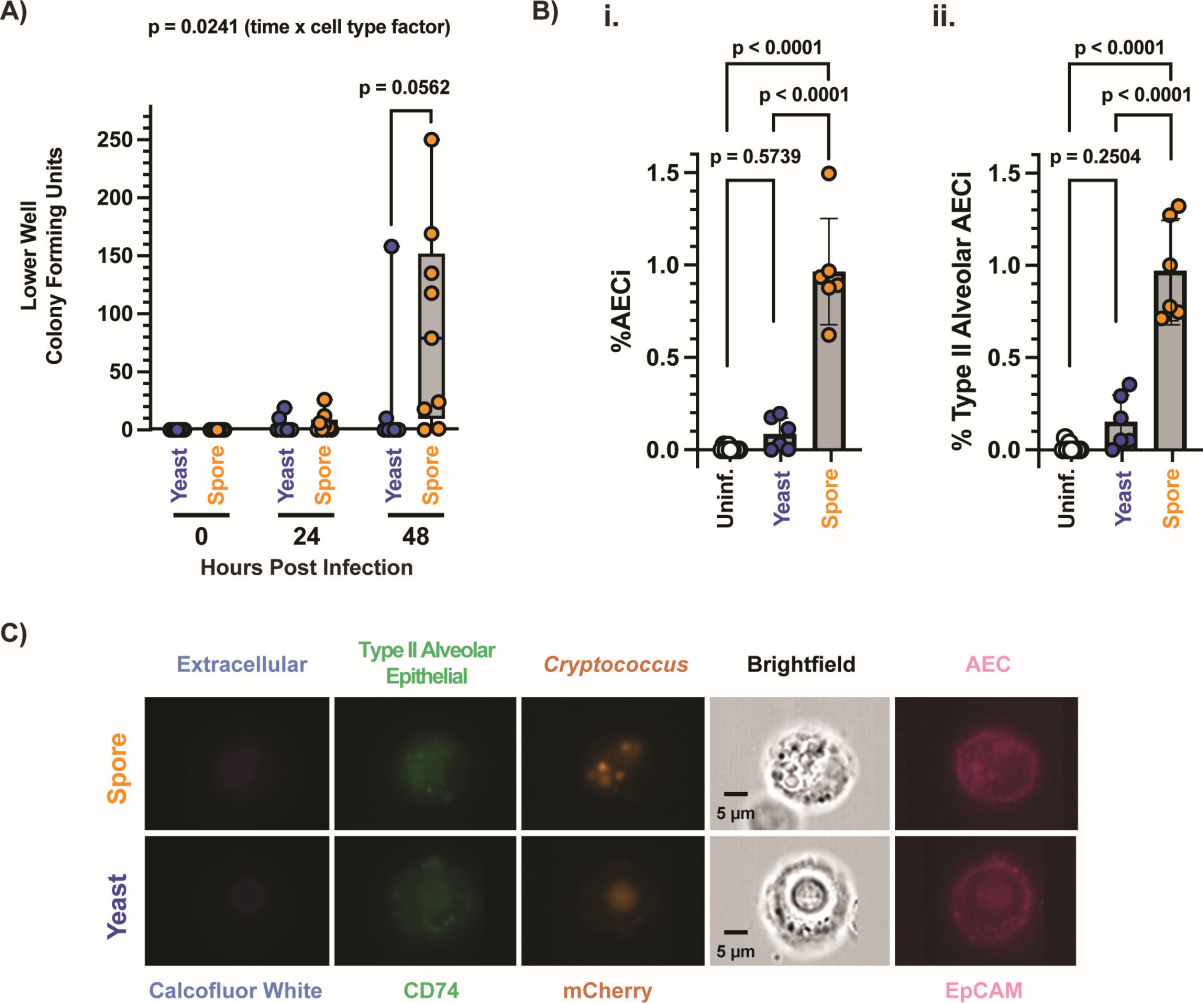
*Cryptococcus* spores preferentially cross AEC barriers and are internalized by AECs in a murine model of infection. (**A**) Lower well colony-forming units at 0, 24, and 48 hours in spore and yeast-infected transwells indicating the crossing of AEC barrier. Statistics from two-way analysis of variance (ANOVA). (**B**) Percentage of (i) AECs (EpCAM^+^) or (ii) type II alveolar epithelial cells (EpCAM^+^, CD74^+^) with intracellular *Cryptococcus* (mCherry^+^, CFW^−^), derived from mouse lungs 8 hours post-intranasal infection with spores or yeast. All statistics were derived from one-way ANOVA across samples. (**C**) Representative imaging flow cytometry panels of *Cryptococcus* cells (both spores and yeast) inside type II airway epithelial cell, 8 hours post-intranasal infection. Ch1, calcofluor white staining differentiates between fungal cells inside and outside AECs. Ch2, CD74 staining is used to identify type II alveolar AECs. Ch4, nuclear localized mCherry tag identifies fungal cells. Ch5: brightfield image of cells. Ch6, EpCAM staining is used to identify AECs.

Spores generated from avirulent *Cryptococcus* yeast (unable to cause disease within 100 days of intranasal infection) preferentially disseminate out of the lung, resulting in disease in intranasal murine models of infection (13). Given the unique ability of spores to enter AECs and subsequently germinate, replicate, and escape, we hypothesized that spore-specific uptake by AECs would enable them to cross epithelial barriers better than yeast. To test this, we monitored *Cryptococcus* crossing across transwells seeded with Calu-3 bronchial-like cell line, which, unlike A549 cells, form tight junctions and impermeable barriers. Spores are internalized by Calu-3 cells but less efficiently than A549 cells (Fig. S3Aiii); thus, confluent Calu-3 cells were infected with either spores or yeast at an MOI of 5. Medium from the lower compartment was plated for colony-forming units at 0, 24, and 48 hours (Fig. 3A). After 48 hours, spores showed preferential crossing compared to yeast, with 88.9% spore-infected wells showing crossing, as opposed to 22.2% of yeast-infected wells. The impermeability of Calu-3 transwell barriers was evaluated by monitoring dextran 70 kDa-Texas Red crossing (Fig. S5A) as a proxy of epithelial barrier integrity, along with transepithelial resistance measurements (Fig. S5B) (31). Provided that the addition of dextran 70 kDa-Texas Red did not alter the preferential *Cryptococcus* spore crossing observed (Fig. S5C), these measurements confirmed the integrity of the transwell barrier beyond 48 hours post-infection. Co-infections with mCh-tagged spores and GFP-tagged yeast also displayed preferential spore crossing, further supporting that spores are better able to cross the lung epithelial barrier than yeast (Fig. S5D).

Given the ability of spores to be internalized by, persist inside, and escape AECs, we evaluated the *in vivo* internalization of *Cryptococcus* by AECs using an intranasal murine model of cryptococcosis. Mice were intranasally infected with 5 × 10^6^ spores or yeast (JEC20mCh/JEC21mCh), and the infection was carried out for 8 hours. This time point was chosen to ensure morphotype-specific characterization because 8 hours is not long enough for spores to germinate into yeast even under nutrient-rich conditions (8, 25). Lungs were harvested, processed, pooled, and differentially stained using our previously established methodology to identify AECs via IFC (Fig. 3B and C) (17). We found that for spore-infected mice, 0.96% of recovered AECs (EpCAM^+^) and 0.97% of recovered type II alveolar AECs (EpCAM^+^, CD74^+^) had internalized spores, with most events having more than one spore (58.6%) and an average stoichiometry of 2.28 spores/AEC_i_ (Fig. S4Ciii). On the other hand, for yeast-infected mice, only 0.09% of recovered AECs and 0.15% of recovered type II alveolar AECs had internalized yeast, with 21.3% having two yeast cells and an average stoichiometry of 1.14 yeast/AEC_i_. These data indicate that spores are able to cross epithelial barriers *in vitro* and get inside AECs *in vivo* better than yeast. These results reveal AECs as a novel intracellular host cell type that *Cryptococcus* can occupy and offer potential alternative mechanisms of spore-specific dissemination and persistence within the host lung.

## DISCUSSION

**Fig 4:**
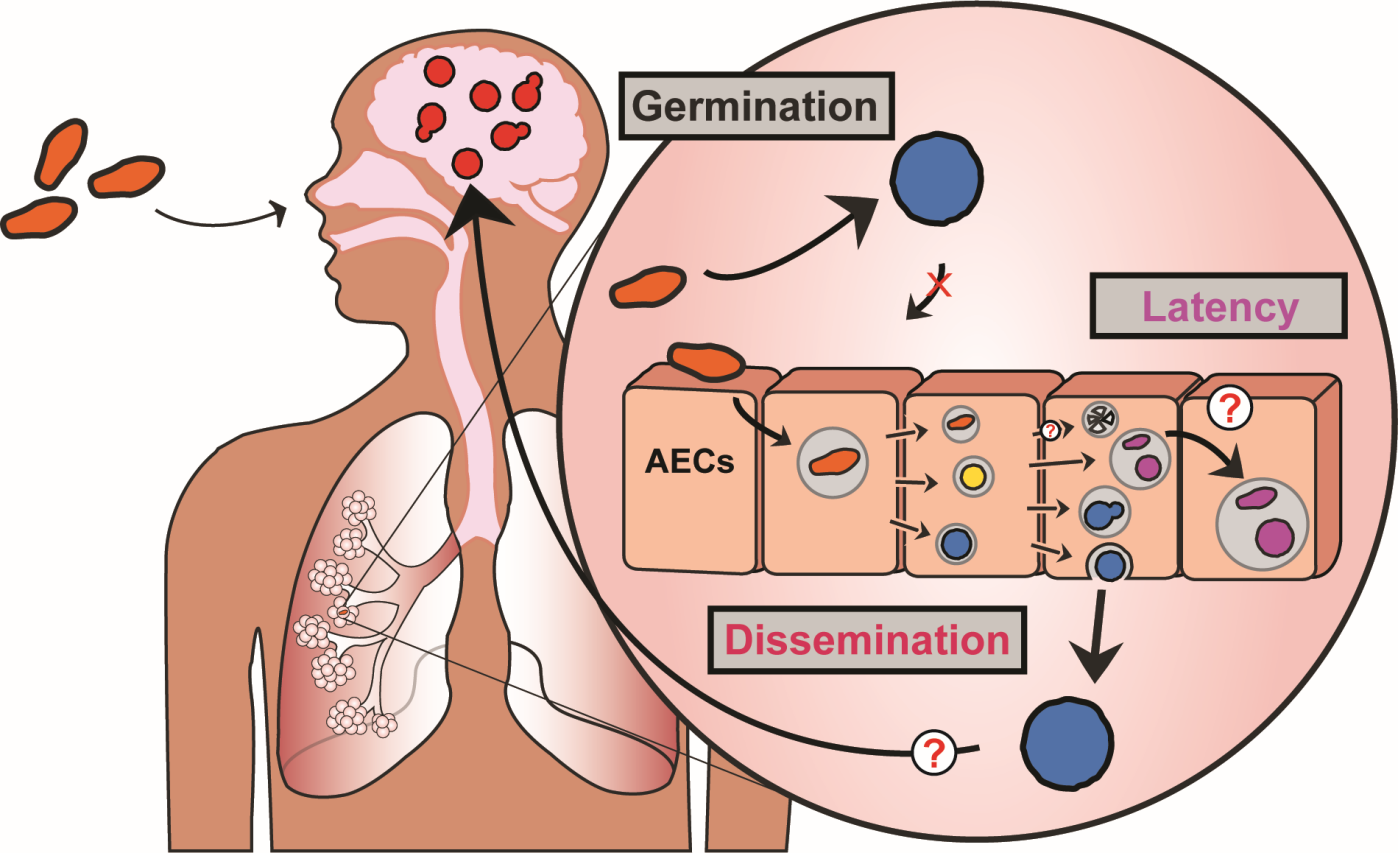
Schematics of our current understanding of *Cryptococcus*-AEC interactions. In this work, we have demonstrated that spores, unlike yeast, are readily internalized by airway epithelial cells. This spore-specific interaction provides a unique intracellular niche where spores can germinate and, once germinated, can replicate, persist, and non-lytically escape. The morphotype-specific ability to enter AECs, survive, and escape provides a viable explanation for *Cryptococcus* spore-specific dissemination out of the lung. The ability of *Cryptococcus* to persist inside AECs, not only as fully germinated yeast but as apparently dormant (ungerminated) spores, provides an unexplored potential mechanism of latency.

*Cryptococcus* spores, along with “desiccated” yeast, are the presumed infectious particles in cryptococcal disease, while vegetatively growing yeast are not generally considered to be the primary infectious propagules due to their large size preventing them from reaching the lower airways. However, likely due to the difficulties associated with working with spores, the vast majority of studies on *Cryptococcus* host-pathogen interactions have been performed with vegetatively growing yeast and not spores. While understanding yeast-host interactions is critical, ignoring other morphotypes prevents us from completely understanding *Cryptococcus* pathophysiology. By neglecting to study these morphotypes, opportunities to develop diagnostics, prevention, and treatments for fungal disease are inevitably missed. In this study, we demonstrate that spores, which have distinct surface properties, interactions with host cells, and disease kinetics to yeast, are internalized by AECs when yeast are not (9–13). While this study did not compare the efficiency of uptake of spores by the epithelial cells and by professional phagocytes, previous work by Walsh et al. compared the association of spores and yeast by professional phagocytes *in vivo*, with the same inoculum (5 × 10^6^ cells/mouse) and similar timing (6 hpi as opposed to 8 hpi), and using different techniques (13). They found that alveolar macrophages showed 18% association with spores as compared to 13.3% for yeast, and dendritic cells showed 1.23% for spores compared to 0.11% for yeast. Our values for uptake of spores and yeast by AECs are similar to those published for dendritic cells and, unsurprisingly, lower than those observed by alveolar macrophages. However, the fold difference of spores within AECs compared to yeast is 21-fold as opposed to 2.7-fold for those of alveolar macrophages, when taking into account stoichiometry. Once inside AECs, spores can germinate, replicate, escape, and/or persist (Fig. 4). Such spore-specific host-pathogen interactions may have major implications for our understanding of both dissemination and latency.

Work by Walsh et al. demonstrated that spores of avirulent yeast (B3501/02) caused disease in an intranasal murine model 50 days after initial infection, while yeast did not (13). Spore-specific virulence was directly linked to the ability of spores to disseminate out of the lungs when yeast could not, with dissemination to the kidneys within 6 days post-infection, and eventually to the brain. Since spores readily germinate within 18 hours in the murine lung, this work suggested that early host-pathogen interactions in the lung dictate spore-specific dissemination and disease. The authors postulated that spore-specific dissemination could result from a Trojan Horse mechanism, where spores hide inside macrophages to get shuttled outside the lung, a prominent hypothesis for yeast dissemination as well. The work demonstrated that spores were more efficient at initially reaching lung draining lymph nodes than yeast, and that trafficking to the lymph nodes was CD11c^+^ dependent. Unfortunately, the authors were unable to evaluate the role of macrophage uptake in further dissemination to other extrapulmonary organs. The data we present here provide an alternative, but not mutually exclusive, hypothesis for the mechanism of dissemination outside the lung. Rather than a macrophage-driven Trojan Horse model, we propose spores may be able to disseminate out of the lung through transcytosis, where spores are internalized by epithelial cells, germinate, and non-lytically escape on the other side of the epithelial barrier. This alternative mechanism is further supported by recent work that identified the infrequent occurrence of “free floating” yeast in the vasculature of murine lungs 7 days post-intranasal yeast infection, consistent with the low levels of internalization by AECs we observed in our murine yeast infections (32). These two mechanisms may even work in tandem, as reported with *Mycobacterium tuberculosis* (*Mtb*), with monocytes containing *Mtb* more efficiently translocating across an epithelial barrier when AECs were also infected with *Mtb* (33). In order to conclusively determine the precise role of both macrophages and AECs in *Cryptococcus* dissemination out of the lung, the molecular mechanisms driving spore-host interactions need to be identified to subsequently modulate spore-host interactions.

The first epidemiological evidence of *Cryptococcus* latency was obtained in 1999 (34). Since this first study, a variety of evidence has supported the idea of latency/persistence in cryptococcal disease (5, 6). Relatively little is known about the mechanisms underlying latency/persistence; however, granulomas have largely been presumed to be the predominant source of latent *Cryptococcus* (35). This idea originally stems from a comparison to *Mtb*, where similar granulomas have been the predominant paradigm for a mechanism of latency (5, 35, 36). However, this has recently been challenged with evidence of *Mtb* DNA being present in non-professional phagocytic lung cells in the absence of tuberculous lesions (36, 37). The frequency and importance of granulomas in cryptococcal disease is unclear, and while granulomas are the presumed latency reservoir in the lung, there may be other mechanisms driving latency. Based on our data, we propose an alternative mechanism where AECs could provide an intracellular environment, shielded from immune cells, in which *Cryptococcus* spores could lie dormant for extended periods of time. The presence of *Cryptococcus* cells within AECs of patients has yet to be investigated.

Dormancy in *Cryptococcus* is poorly understood, and the morphological state of *Cryptococcus* during latency is unknown. Importantly, spores are the classical example of a dormant particle, and for *Cryptococcus,* these are exclusively produced in the environment, not inside the host. Spores show distinct nutrient utilization to yeast, but the molecular mechanisms that control the escape from dormancy still require characterization (8, 11). It is possible that spores, being a fundamentally dormant morphotype, are more readily able to remain dormant (or re-establish dormancy) in a host than yeast. The identification of AECs as a spore-specific intracellular environment, where spores can persist and germinate to varying degrees, provides a novel alternative for understanding latency and persistence in cryptococcal disease. It is important to note that our evaluation of intracellular persistence was limited to 5 days post-internalization *in vitro*, and that the duration that *Cryptococcus* can remain inside AECs and the implications of these interactions in disease kinetics remain to be investigated. A growing body of evidence shows an important role for AECs in the antimicrobial potency of the lung, where the interactions of these non-professional phagocytes with pathogens can be either a benefit or detriment to the host (15, 16). This likely depends on both pathogen factors and host predispositions. The role of AECs in cryptococcal disease has largely been ignored due to the lack of internalization of the yeast morphotype; it will be interesting to understand if other understudied morphotypes present in the lung, such as viable but non culturable cells, seed cells, and titanides, could interact similarly with AEC as spores do. This study expands on the already long list of differences between *Cryptococcus* spores and yeast and points toward AECs as a novel intracellular environment for the field to consider when studying *Cryptococcus* disease kinetics and potential therapeutics.

## MATERIALS AND METHODS

### Fungal strains and strain manipulation

*Cryptococcus neoformans* serotype D (*deneoformans*) strains JEC20-mCh (CHY4031), JEC21-mCh (CHY4028), JEC20-GFP (CHY3955), JEC21-GFP (CHY3952), and *Cryptococcus neoformans* serotype A strain KN99α-mCh were handled using standard techniques and media as described previously (13, 38, 39). *Cryptococcus* spores were isolated from cultures as described previously (9). Briefly, yeast of both mating types (JEC20-mCh and JEC21-mCh) were grown at 30°C for 2 days on YPD agar, resuspended in 1× phosphate-buffered saline (PBS) (Sigma-Aldrich: D8537), mixed in a ratio of 1:1, and intermittently spotted onto dried V8 pH 7.0 agar plates. Plates were incubated for 7 days at 25°C in the dark, and crosses were resuspended in 75% Percoll (Sigma-Aldrich: GE17-0891-01) in 1× PBS for separation via gradient centrifugation (2,110 × *g*, 25 min). Spores were recovered, counted using a hemocytometer, and assessed for purity by visual inspection. Germinated spores (JEC20×21 yeast) were generated by growing purified spores at 30°C for 3 days on YPD agar.

To produce mCherry-tagged KN99“a”×α spores, a KN99“a”-mCh strain was derived by mating KN99α-mCh yeast with KN99“a” yeast on Murashige and Skoog pH 5.8 agar plates for 2 weeks. Spores were grown out for single colonies on YPD, screened for fluorescence to confirm mCh signal, and evaluated for mating ability with KN99α to identify “a” mating type. For infections, KN99“a”×α spores were derived as described above, but a 90% Percoll was used for gradient centrifugation, and due to 65% spore:yeast purity, inoculum was adjusted to account for yeast presence.

### Epithelial cell culture

The cell lines used in this study were A549, human pulmonary carcinoma epithelial cells (ATCC: CCL185), and Calu-3, human pulmonary adenocarcinoma epithelial cells (ATCC: HTB-55). A549 cells were maintained in Dulbecco′s modified Eagle′s medium (DMEM) (Sigma-Aldrich: D5796) supplemented with 10% Gibco fetal bovine serum (FBS) (Thermo Fisher Scientific: A5256801) and 1% penicillin/streptomycin cocktail (Sigma-Aldrich: P0781) (DMEMs). Calu-3 cells were maintained in DMEM/F12 (Thermo Fisher Scientific: 11320033) supplemented with 10% FBS and 1% penicillin/streptomycin cocktail (DMEM/F12s). Primary human AECs were purchased from Lonza (healthy = 2547, TAN 43140 and 40273) following isolation from the distal portion of the human respiratory tract in the 1 mm bronchiole area. Primary AECs were maintained in Small Airway Epithelial Cell Growth Medium (Promocell) and used for infections within the ninth passage. All cells were grown and maintained at 37°C, 5% CO_2_, with all passaging and seeding performed with trypsin-EDTA (Sigma-Aldrich: T3924).

### Flow cytometry

Differential fluorescence IFC was performed following published methodology (23). Briefly, confluent monolayers of A549 (or primary cells) cells across triplicate wells of six-well plates (Greiner: 657160) were infected with either spores (JEC20-mCh×JEC21-mCh, KN99“a”-mCh×KN99α-mCh), yeast (JEC20-mCh+JEC21-mCh, KN99α-mCh, KN99“a”+KN99α), or germinated spores (JEC20×21 yeast) at varying MOIs (0.167, 1, or 5) for different times (6 hours, 24 hours). All experiments had uninfected controls processed identically as negative controls and for gating purposes. For yeast/spore co-infections, A549 cells were infected at an MOI of 0.167 (per morphotype) with either spores (JEC20-mCh×JEC21-mCh or JEC20-GFP×JEC21-GFP), yeast (JEC20-mCh+JEC21-mCh or JEC20-GFP + JEC21-GFP), or combinations of two fluorescently distinct cell types. For intracellular persistence evaluation, after 6 hours of incubation, A549 cells were reseeded to attain confluency 120 hours later (effectively 126 hpi). At the end of the infections, A549 cells were trypsin digested and collected via centrifugation at 500 × *g*. Cells were incubated in 500 µL of binding buffer, 1× (5 µL) annexin V-FITC (Abcam: ab14085), 100 nM TO-PRO-3 (Thermo Fisher Scientific: T3605), and 8 µg/mL CFW (Sigma-Aldrich: F3543) for 5 min, centrifuged, and washed with 1× PBS. Cells were resuspended and evaluated on an Amnis ImageStream MkII. For each sample, 5,000 single cells in focus were analyzed across three independent acquisitions in biological triplicate. Analysis was performed for the identification of internalization and host cell death using IDEAS software according to a previously defined analysis pipeline (24). Uninfected A549 samples were used for background subtraction, and all samples were normalized based on infectious inoculum as determined by triplicate CFU counts. Visualization of internalization events was performed on both IDEAS software and Fiji/Imato quantify stoichiometry and germination. During data export, IDEAS adds extra space around cells for visualization, which can make partially captured cells appear cropped. *Cryptococcus* germination quantification was performed by evaluation of area and aspect ratio of internalized *Cryptococcus* cells following previously established cutoffs (spores: area < 7.44 µm^2^, aspect ratio < 0.8; yeast: area > 7.84 µm^2^, aspect ratio > 0.8) as defined by Barkal et al. (25) across all biological replicates, with technical replicates acquired for internalization evaluation.

FACS (using a BD Influx Cell Sorter) was used to sort persistent *Cryptococcus* 126 hours post-infection with spores. Infections and processing were performed as detailed above, and single A549 cells containing intracellular *Cryptococcus* (mCh^+^/CFW^−^) were sorted into wells of 96-well plates containing YPD for three technical replicates for each of three infections (biological replicates). Plates were incubated at 30°C for 1 week, and wells were visualized under a microscope and scored for the presence of *Cryptococcus* growth.

### Microscopy

A549 cells were seeded into 24-well glass bottom plates (Greiner: 662892) and, when confluent, infected with JEC20-mCh×JEC21-mCh spores at an MOI of 1. After 6 hours, medium was removed and cells were stained with 8 µg/mL CFW for 5 min, washed with 500 µL 1× PBS, and resuspended in Gibco FluoroBrite DMEM (Thermo Fisher Scientific: A1896701) supplemented with 10% FBS and 1% penicillin/streptomycin cocktail. All images were acquired on a Nikon Eclipse Ti Inverted Microscope, using a Nikon CFI Plan APO λ 40× objective and Hamamatsu ORCA-Flash4.0 LT C11440 camera, at 37°C and 5% CO_2_ for 20 hours after staining. Images were acquired across three biological replicates, three wells per replicate (10 positions/well) in brightfield, 400 nm (100 ms exposure) and 550 nm (300 ms exposure). For each biological replicate, 240–348 internalization events (determined by CFW^-^ signal at 6 hours post-infection) were monitored hourly from 6 to 26 hours. For each internalized *Cryptococcus* event, germination (determined by size/shape), replication (determined by the addition of intracellular *Cryptococcus* cells, i.e., budding), rapid replication (determined by the number of intracellular *Cryptococcus* cells at least tripling by 26 hours and visual ballooning), vacuole formation (determined by a large ring surrounding intracellular *Cryptococcus* cells), and non-lytic escape (determined by intracellular *Cryptococcus* cells floating away from host cells) were evaluated by microscopy and quantified.

Germination of spores was evaluated across 24 hours in tissue culture conditions (supplemented DMEM, 37°C, 5% CO_2_), and images were acquired on a Nikon Eclipse Ti Inverted Microscope, using a Nikon CFI Plan Fluor ELWD 20× objective and Hamamatsu ORCA-Flash4.0 LT C11440 camera. Germination was quantified using the previously published quantitative germination assay and custom algorithms (24, 25).

### Transwell crossing

Transwell crossing experiments were performed by seeding Falcon permeable supports for 12-well plates with 8.0 µm transparent PET membranes (Corning: 353182) and associated companion plates (Corning: 353503) with Calu-3 cells at 1 × 10^6^ cells/well. Cells were allowed to reach confluency (8–12 days) as determined by a resistance measurement of >900 ohms using a Milipore Millicell ERS-2 Epithelial Volt-Ohm Meter and Milicell ERS-22 Adjustable Electrode Set (MERSSTEX03). Each upper well of the transwell was infected with either spores or yeast (MOI 5), and crossing was evaluated by plating lower well volumes onto YPD plates at 0, 24, and 48 hours post-infection. YPD plates were incubated for 3 days, and the total number of colonies/well/day was quantified. The impermeability of the barrier was further confirmed by lack of fungal crossing following infection, and resistance was monitored across all time points. Crossing was evaluated for nine replicates of both spore- and yeast-infected wells.

In addition to the transepithelial resistance measurements, supplemental experiments based on previously published methodology (31) were performed using 70 kDa dextran-Texas Red (Thermo Fisher: D1830), which was added to the upper wells at a concentration of 0.1 µM. Dextran crossing was monitored by evaluating the fluorescence (595/615 nm) of the lower well media every 24 hours post-infection. Fungal crossing experiments were repeated, using the methodology above, in the presence of dextran with spores (JEC20×21-mCh), yeast (JEC20+21-GFP), and with co-infections of both. Colonies from co-infection experiments were evaluated for either mCh or GFP signal to determine if they were spore-derived or yeast-derived *Cryptococcus* crossing events, respectively.

### Murine infection, lung processing, and staining for imaging flow cytometry

Murine infections were performed under UK Home Office Project License PBE275C33 at the Biomedical Service Unit, University of Birmingham. Mice were housed in individually ventilated cages under specific-pathogen-free conditions, with access to standard chow and drinking water *ad libitum* under a 12 hour light/dark cycle at 20°C–24°C and 45%–65% humidity. Spores (JEC20-mCh×JEC21-mCh) and yeast (JEC20-mCh+JEC21-mCh 50:50 mix) were enumerated using a hemocytometer and diluted in 1× PBS. Three groups of 8- to 10-week-old female C57BL/6NCrl mice (Charles River) were left uninfected (six mice) or intranasally infected with spores (four mice) or yeast (four mice) at a final dose of 5 × 10^6^ cell/mouse. Mice were humanely euthanized by cervical dislocation at 8 hours post-infection, and lungs were perfused with 2 mL of 0.1 mg/mL dispase (Sigma-Aldrich: D4693) in HANKS buffer (Sigma-Aldrich: H9269) directly into trachea. Lungs were processed as previously described for digestion and typing of AECs (17). Cell suspensions from dissociated infected murine lungs were incubated with 1 µg/mL Ep-CAM (CD326) PE/Cy7 antibody (BioLegend, 118215) and 1 µg/mL CD74 FITC antibody (Santa Cruz Biotechnology, sc-6262-FITC) for 25 min at 4°C, followed by 8 µg/mL CFW for 5 min. Samples were centrifuged at 500 × *g* for 3 min and resuspended in 100 µL of 1× PBS + 2% FBS + 2 mM EDTA prior to evaluation on an Amnis ImageStream MkII. For each pooled sample (three uninfected, two yeast-infected, and two spore-infected), three acquisitions of 3,000–4,000 EpCam^+^ single cells in focus were taken as technical replicates. Data analysis was performed using the IDEAS software and the pipeline previously described, using single-stain controls for gating purposes.

### Statistical analysis of data

GraphPad Prism was used to interpret data, and *P*-values were calculated through unpaired *t*-tests or ordinary one-way or two-way analysis of variance and correlation analyses as specified in the figure legends. Error bars show standard deviation.

## SUPPLEMENTAL MATERIAL

Figure S1 mbio.01831-25-s0001.tif Additional imaging flow cytometry controls validating spore-specific uptake by AECs by *Cryptococcus*.

Figure S2 mbio.01831-25-s0002.tif Germination of spores in tissue culture conditions.

Figure S3 mbio.01831-25-s0003.tif Relative *Cryptococcus* internalization and host cell damage.

Figure S4 mbio.01831-25-s0004.tif Intracellular *Cryptococcus* stoichiometry.

Figure S5 mbio.01831-25-s0005.tif Additional controls for preferential crossing of spores across AEC (Calu-3 cells) barrier.

Figure S6 mbio.01831-25-s0006.tif Shape of intracellular *Cryptococcus* persisting 126 hpi.

ASM does not own the copyrights to Supplemental Material that may be linked to, or accessed through, an article. The authors have granted ASM a non-exclusive, world-wide license to publish the Supplemental Material files. Please contact the corresponding author directly for reuse.
