# Supplementary figures and images for: Cryptococcus displays spore-specific uptake by alveolar epithelial cells
Source: mBio. 2025 Oct 22;16(11):e01831-25. doi: 10.1128/mbio.01831-25 (PMC12607909; doi:10.1128/mbio.01831-25)

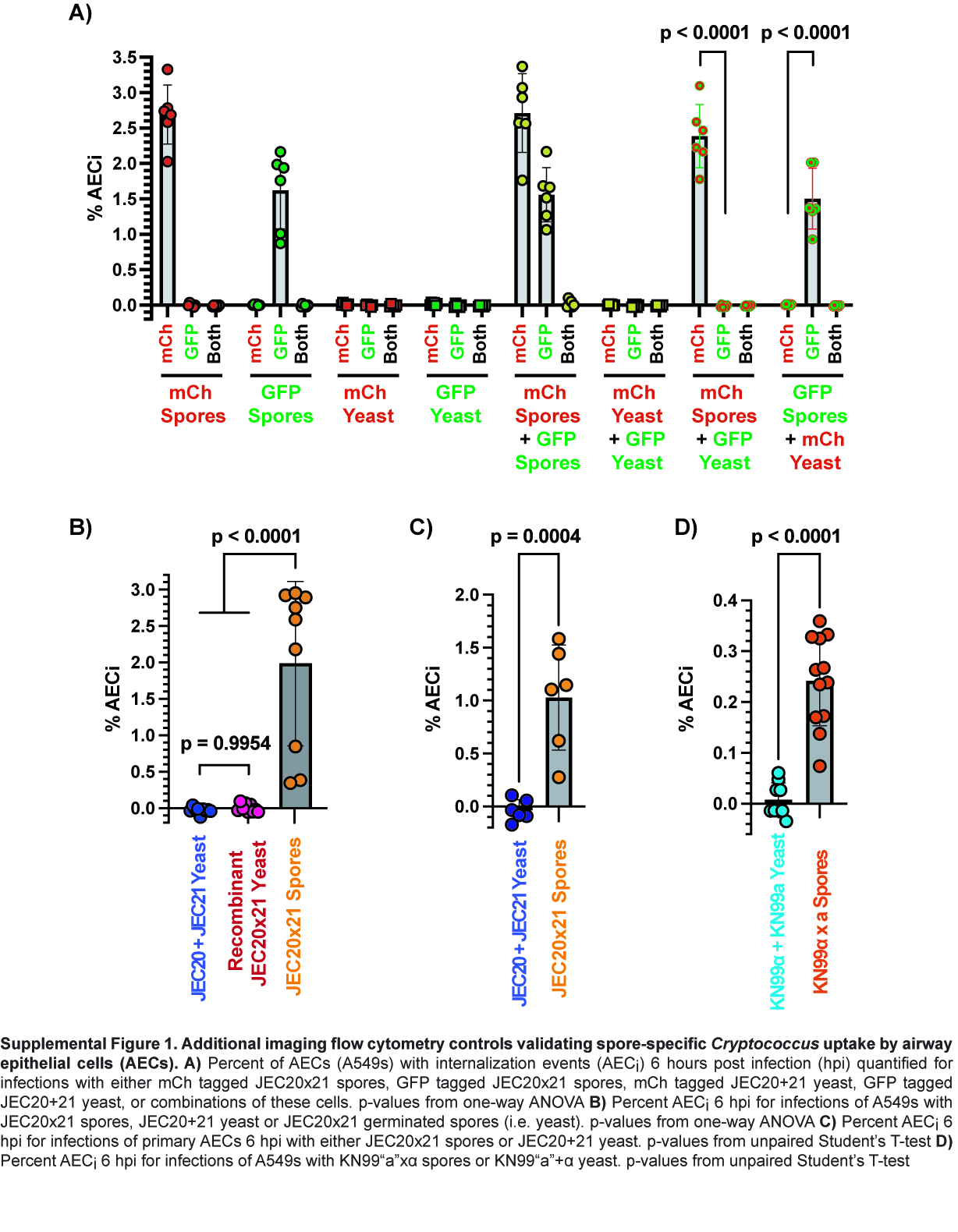

Supplement: Figure S1 — Additional imaging flow cytometry controls validating spore-specific uptake by AECs by Cryptococcus. [file mbio.01831-25-s0001.tif]

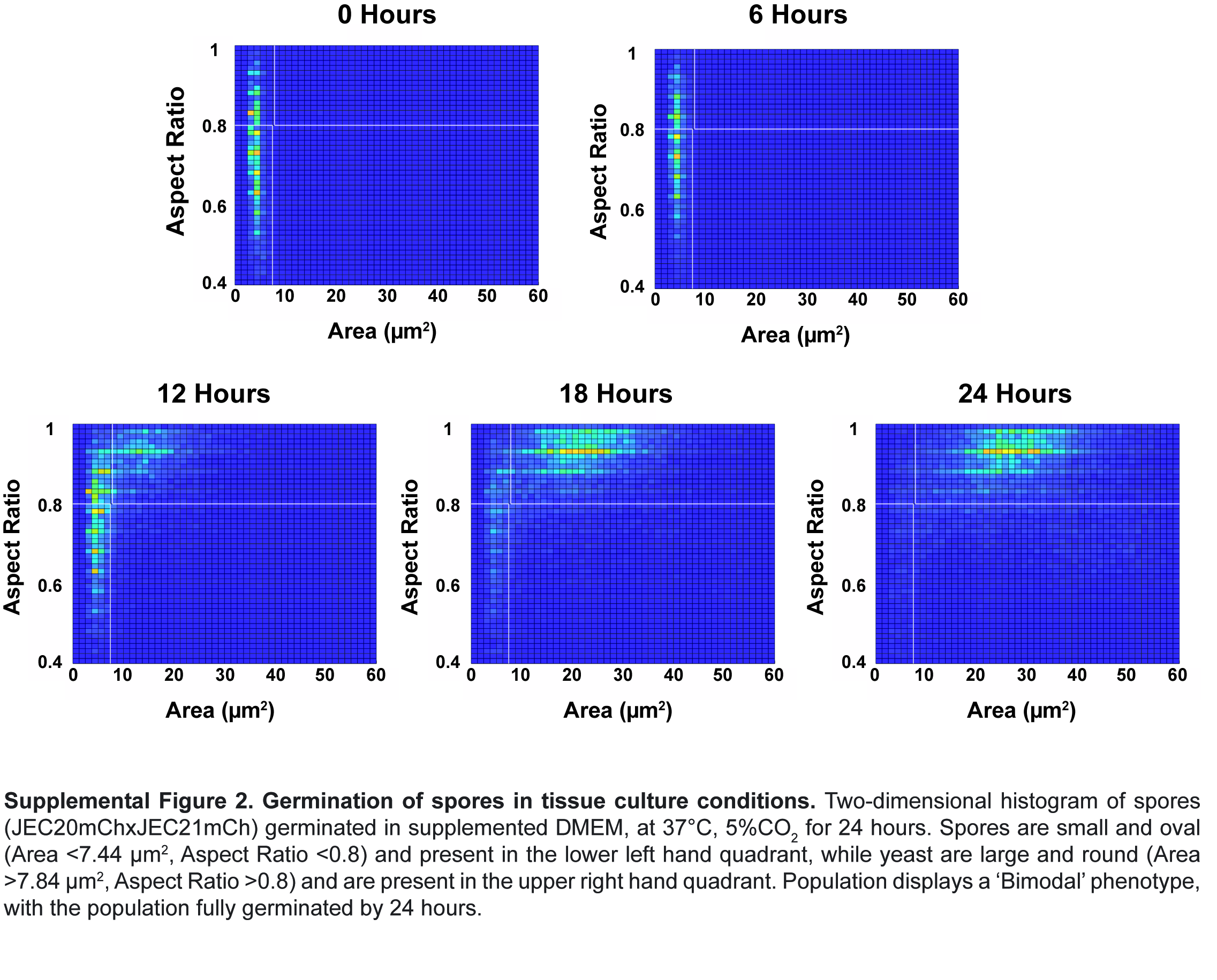

Supplement: Figure S2 — Germination of spores in tissue culture conditions. [file mbio.01831-25-s0002.tif]

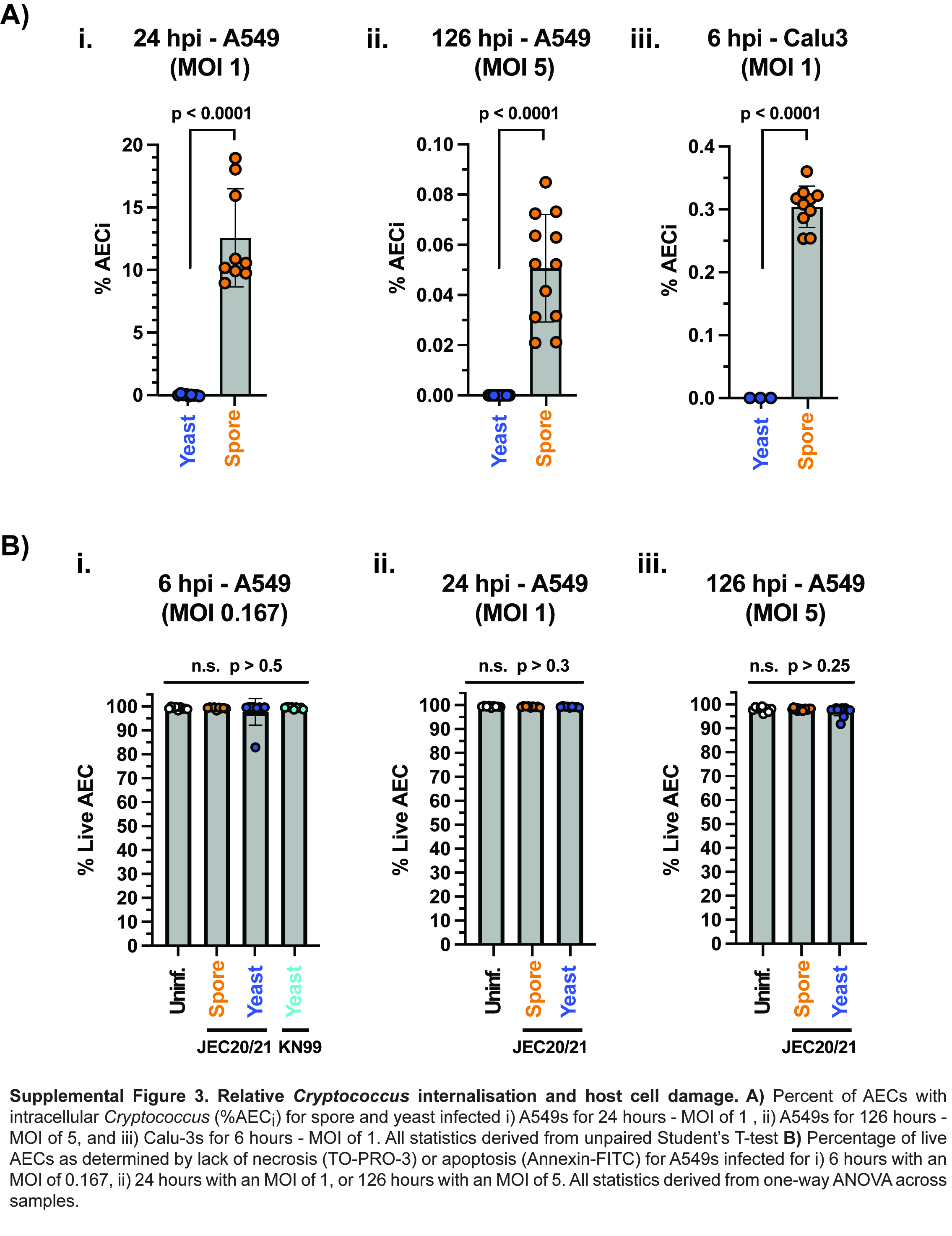

Supplement: Figure S3 — Relative Cryptococcus internalization and host cell damage. [file mbio.01831-25-s0003.tif]

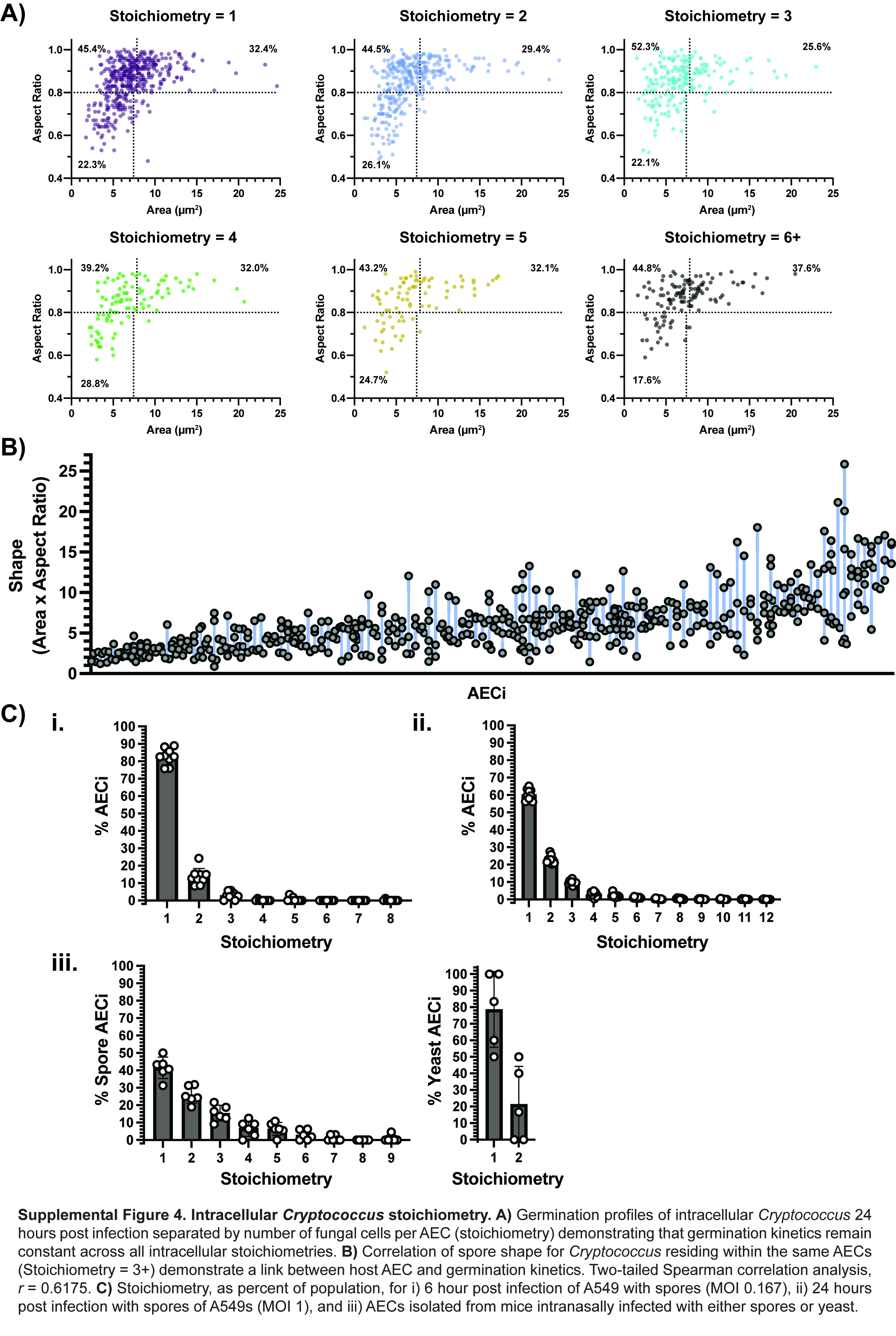

Supplement: Figure S4 — Intracellular Cryptococcus stoichiometry. [file mbio.01831-25-s0004.tif]

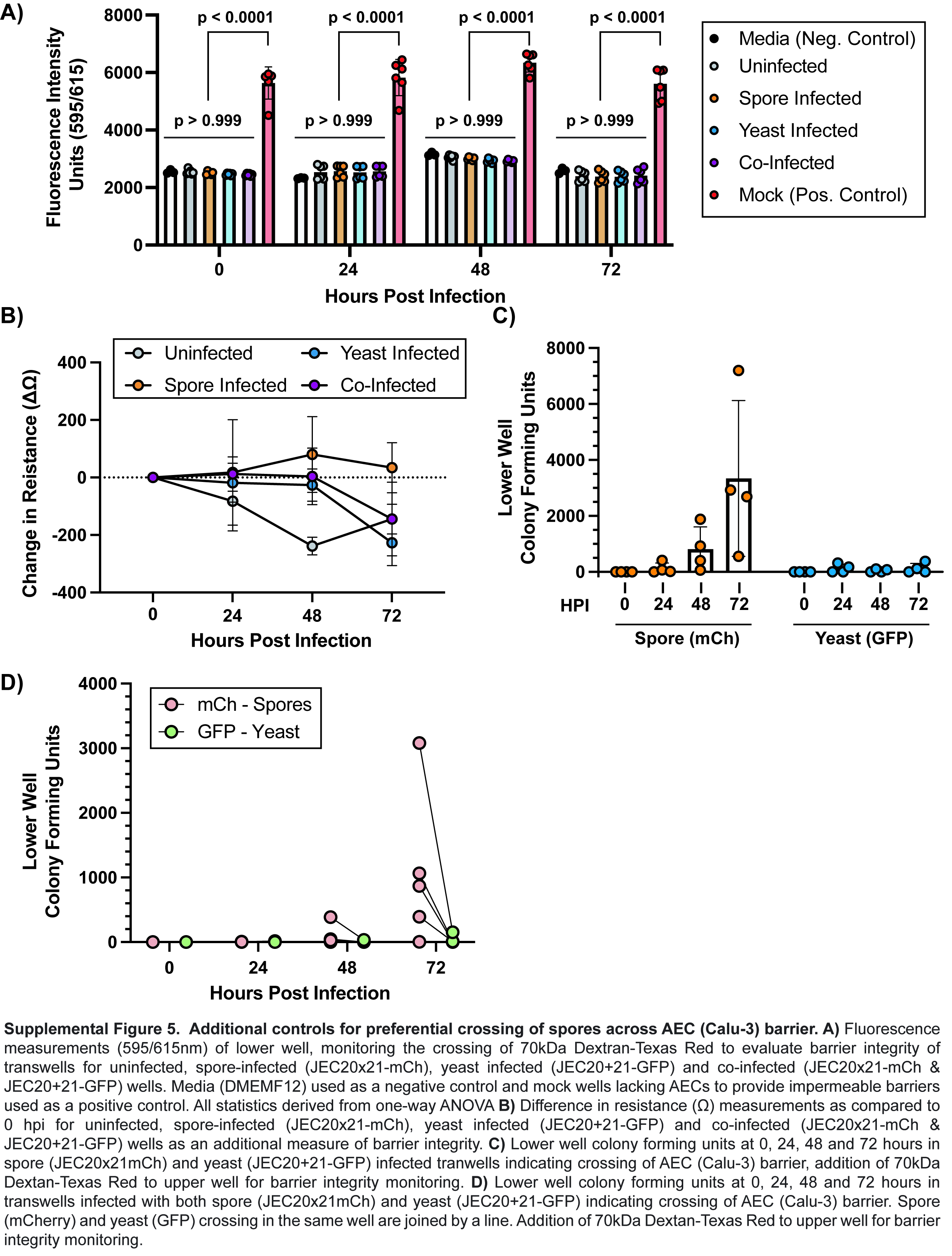

Supplement: Figure S5 — Additional controls for preferential crossing of spores across AEC (Calu-3 cells) barrier. [file mbio.01831-25-s0005.tif]

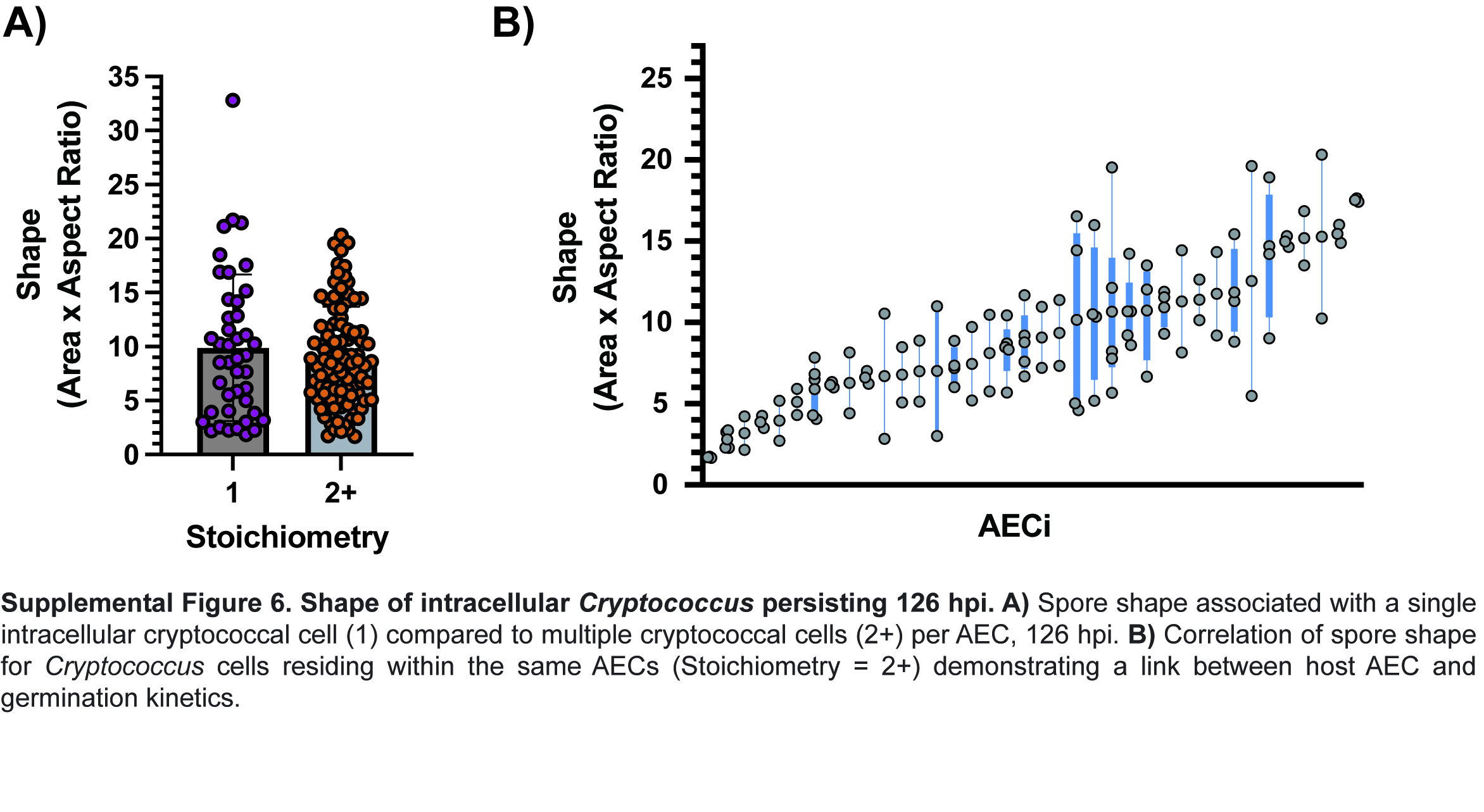

Supplement: Figure S6 — Shape of intracellular Cryptococcus persisting 126 hpi. [file mbio.01831-25-s0006.tif]
